# Communication between the gut microbiota and peripheral nervous system in health and chronic disease

**DOI:** 10.1080/19490976.2022.2068365

**Published:** 2022-04-28

**Authors:** Tyler M. Cook, Virginie Mansuy-Aubert

**Affiliations:** Department of Cell and Molecular Physiology, Loyola University Chicago, Maywood, IL, USA

**Keywords:** Gut microbiota/ microbiota metabolites/PNS/neuronal sensing/obesity

## Abstract

Trillions of bacteria reside within our gastrointestinal tract, ideally forming a mutually beneficial relationship between us. However, persistent changes in diet and lifestyle in the western diet and lifestyle contribute to a damaging of the gut microbiota-host symbiosis leading to diseases such as obesity and irritable bowel syndrome. Many symptoms and comorbidities associated with these diseases stem from dysfunctional signaling in peripheral neurons. Our peripheral nervous system (PNS) is comprised of a variety of sensory, autonomic, and enteric neurons which coordinate key homeostatic functions such as gastrointestinal motility, digestion, immunity, feeding behavior, glucose and lipid homeostasis, and more. The composition and signaling of bacteria in our gut dramatically influences how our peripheral neurons regulate these functions, and we are just beginning to uncover the molecular mechanisms mediating this communication. In this review, we cover the general anatomy and function of the PNS, and then we discuss how the molecules secreted or stimulated by gut microbes signal through the PNS to alter host development and physiology. Finally, we discuss how leveraging the power of our gut microbes on peripheral nervous system signaling may offer effective therapies to counteract the rise in chronic diseases crippling the western world.

## 1. Introduction

**Figure 1.**
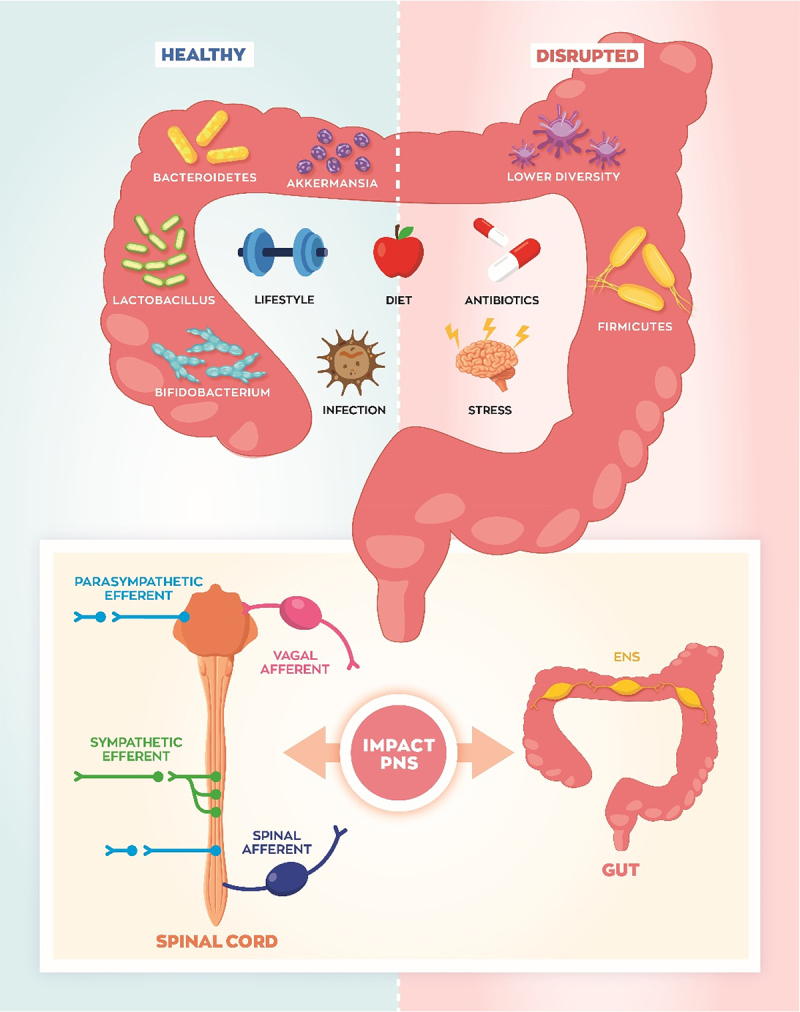
The gut microbiome composition is established at birth and is continually shaped by environmental factors such as lifestyle, diet, antibiotic use, infection, and stress. Generally, healthy individuals have a highly diverse microbiome, enriched in *Bacteroidetes, Lactobacillus, Bifidobacterium*, and *Akkermansia*. In chronic disease, microbiome diversity is often reduced and *Firmicutes* are expanded. The composition of gut microbes drastically impacts the peripheral nervous system (PNS) development and function. Vagal and spinal afferent (sensory) neurons relay microbial signals to the brain, and autonomic output is carried by sympathetic and parasympathetic efferent neurons. Enteric neurons form their own network in the gut, and they are ideally positioned to detect gut microbe signaling and reflexively alter gastrointestinal functions. The afferent, efferent, and enteric nervous systems are interconnected to respond to gut microbe signaling and cooperatively control a variety of homeostatic functions such as digestion, immunity, and visceral perception.

The gut microbiota consists of a dynamic community of trillions of bacteria, archaea, virus, and fungi, which is primarily established at birth from interaction with the mother’s microbiota. Environmental factors such as geographical location, diet, antibiotic exposure, and infection continue to gradually shape microbiota composition during first few years of life.^1^^,2^ The bacterial community that ultimately colonizes the gut should ideally be evolved to symbiotically function with the host, aiding in digestion and proper immune response. However, dramatic changes in diet and lifestyle within the last century have contributed to the explosion of non-communicable diseases such as obesity, diabetes, nonalcoholic fatty liver disease, and irritable bowel syndrome (IBS), and a disconnection between the gut microbiota and the host physiology likely contributes.^3,4^ With increased processing of foods and usage of antibiotics, preservatives, and other additives, our diet is apparently no longer suited for our gut bacteria, and over time the western diet may actively disrupt the balance and diversity of microbes within our gut^5^ (Figure 1). Additionally, insults such as infection disrupt the gut microbiome composition predisposing individuals to gastrointestinal (GI) issues.^6^

Dr. George Fox and Dr. Carl Woese were among the first to leverage the 16S ribosomal RNA gene as a unique marker to sequence the microbiome composition.^7^ Subsequent advances in genomic sequencing technology sparked an explosion of studies proposing associations between various bacteria and host processes or diseases. In the past two decades thousands of studies have aimed to identify microbial signatures, genes, or key species which underly the increased prevalence of various diseases, or to identify potential bacteria with therapeutic potential. Elegant studies performed by Jeff Gordon’s research team identified associations between the *Firmicutes* and *Bacteroidetes* phyla and adiposity, postulating the *Firmicutes*/*Bacteroidetes* ratio as a putative signature of the obese microbiome^8–10^ (Figure 1). They showed that transplanting bacteria from genetically obese mice (ob/ob) caused wild-type littermates to gain more fat mass, as compared to mice transplanted with a lean wild-type microbiota.^8^ Accompanying studies by Gordon’s group found that the microbiota of obese mice and overweight humans is comprised of higher percentage of *Firmicutes*.^10^ Lean individuals displayed higher abundance of *Bacteroidetes*, and as people lost weight via different diet regiments the population of *Bacteroidetes* expanded.^9^ However, associations between the *Firmicutes*/*Bacteroidetes* ratio and body weight have not been consistently supported.^11^ *Prevotella* species ferment fiber and often correlate with healthy metabolic outcomes and reduced inflammation,^12–14^ however there are conflicting findings on whether or not *Prevotella* are expanded in IBS patients.^15,16^ Reduced bacterial diversity, richness, and stability are often reported in obesity and IBS,^10,17^ but overall, identifying reliable microbial disease signatures has been a major challenge.^11^ Nonetheless, therapeutic candidates such as *Akkermansia*,^18–20^ *Lactobacillus*,^21,22^ *Bifidobacterium*,^23–25^ and *Dysosmobacter welbionis*^26^ have emerged as promising targets for obesity or IBS.

**Figure 2.**
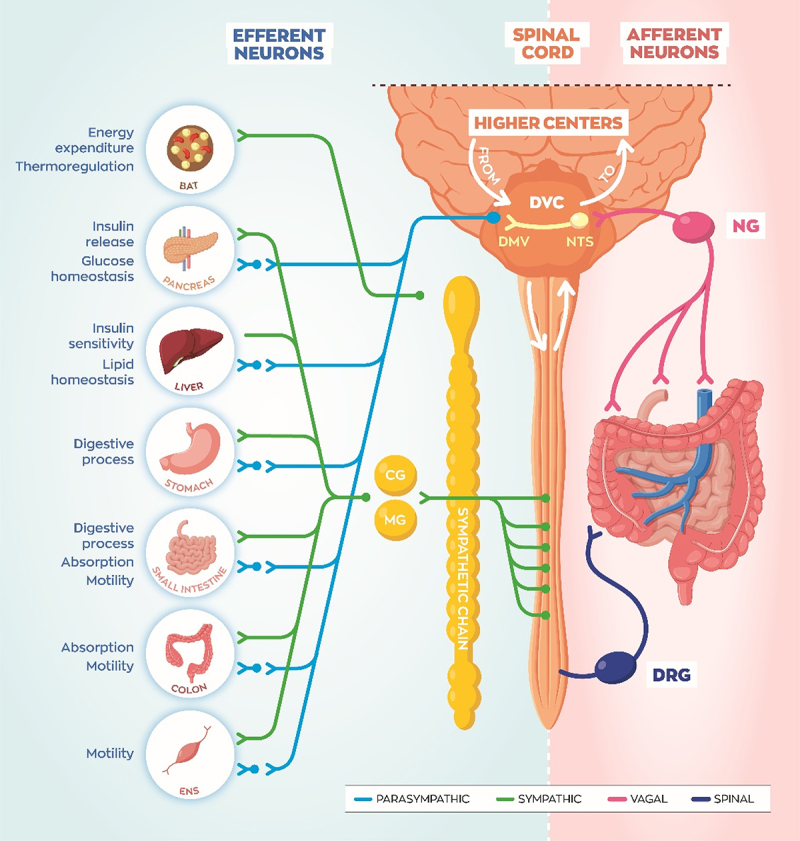
Spinal and vagal sensory neurons innervate the gastrointestinal (GI) tract and portal vein probing activities of the gut microbiota. Vagal sensory neurons with cell bodies in the nodose ganglia (NG) project to the nucleus tractus solitarius (NTS). The NTS and dorsal motor vagus (DMV), as well as the area postrema, comprise the dorsal vagal complex (DVC) in the hindbrain. Spinal sensory neurons with cell bodies in the dorsal root ganglia (DRG) project into the spinal cord to relay visceral signals to the brain. The vagal efferent system is comprised of long preganglionic neurons projecting out from the DMV connecting with short postganglionic neurons which then reach target organs. Short sympathetic efferent neurons leave the spinal cord and connect with postganglionic neurons in the sympathetic chain or discrete peripheral ganglia such as the celiac ganglia (CG) and mesenteric ganglia (MG). Sympathetic projections to brown adipose expends energy to produce heat (thermogenesis) during cold exposure or after a meal. Sympathetic innervation of pancreas and liver mobilizes glucose for energy, while GI innervation halts digestion, during a “fight or flight” state of arousal. Parasympathetic efferent projections generally oppose these actions to return the body back to baseline, during an internal state of “rest and digest”.

Mechanistically there are three general ways in which the gut microbiota and host can communicate to regulate metabolism and digestion: immunological, hormonal, and neuronal.^26–29^ Interestingly, there is a high degree of intercommunication between these modalities within the gut, and the peripheral nervous system (PNS) participates in the immunological and hormonal responses to gut bacterial biochemical processes. As shown in Figures 1 and 2, gut microbe signals can be “sensed” via vagal^30,31^ and spinal neurons,^32^ integrated in the brainstem and hypothalamus, and this ultimately influences the efferent signals to peripheral organs. Increasing efforts have been placed on understanding the molecular interactions between the gut microbiota and host PNS to identify causes of diseases, such as obesity and IBS, and to find novel therapeutic targets. Several recent studies manipulating the gut microbiota composition illustrate the importance of the interaction between gut microbes and the PNS in regulating host physiology. Antibiotics treated mice exhibit reduced innervation and disrupted excitability of enteric neurons, which contributes to slowed intestinal transit time and reduced motility.^33–36^ Maternal gut microbiota dramatically influences the development and maturation of the PNS, influencing host physiology.^33–38^ Furthermore, different components of the peripheral nervous system express nuclear and G-protein coupled receptors (GPCRs) which allow these neurons to “sense” gut microbe signaling.

In this review we will focus on describing how the PNS serves as a relay between the gut microbiota and the host to regulate digestion, gastrointestinal function, and energy balance. We will begin by detailing the general function and anatomy of the peripheral nervous system. We will then highlight the key microbial signaling molecules that signal through the PNS to regulate physiology. Finally, we will discuss promising therapeutic targets for various chronic diseases and comorbidities. Furthering our mechanistic understanding of these interactions could provide insights into disease pathology and identify new treatment strategies.

## 2. Function and anatomy of the peripheral and enteric nervous systems

Neuronal transmission allows for nearly instantaneous processing of sensory input or generation of motor output. This rapid signaling of peripheral neurons in the gut is critical for homeostatic mechanisms such as GI motility, secretion, and even immune response modulation.^39^ The peripheral nervous system (PNS) consists of vagal and spinal sensory (afferent) neurons, autonomic motor (efferent) neurons, and enteric neurons (Figure 2). Afferent neurons send information from the periphery to the brain or spinal cord, while efferent neurons project out from the central nervous system (CNS) to peripheral organs. Classifying by anatomical distribution, the twelve cranial nerves project from the brain/brainstem and spinal nerves from the spinal cord. The autonomic system is divided into sympathetic, parasympathetic, and enteric nervous systems (ENS).

### 2.1 Afferent neurons

As illustrated in Fig.1 and 2, vagal and spinal afferent neurons innervate the digestive tract, monitoring mechanical, chemical, thermal, and nociceptive signals related to the diet and microbiota.^40–45^ It is important to note that some enteric neurons are also characterized as afferent and they are labeled as “intrinsic”, while spinal and vagal neurons which originate outside of the gut are “extrinsic”. Vagal afferent neurons transmit signals up from the viscera, their cell bodies are located in the nodose ganglia (NG), and they synapse into the solitary nucleus (NTS) in the brainstem (Figure 2). The NTS integrates vagal afferent signals and relays the information up to higher brain regions such as the hypothalamus, or reflexes back down to the dorsal motor nuclei of the brainstem where vagal efferent neurons project out to effector organs.^46^ Spinal neurons, with cell bodies in the dorsal root ganglia (DRG), project into the dorsal horn of the spinal cord. These signals are relayed up to the brain and integrated, or they induce reflex activation of motor neurons which may bypass the brain. The spinal nerves can be subdivided into 5 divisions: cervical, thoracic, lumbar, sacral, and coccygeal, based on their projections into and out of the vertebrae.

### 2.2 Efferent neurons and target tissues

The afferent system provides critical information to the CNS, which integrate the visceral information and produces an effect on peripheral organs via autonomic efferent fibers. Although oversimplified, the sympathetic and parasympathetic efferent neurons are generically segregated based on functions aiding in stress responses (fight or flight) or returning to baseline (rest and digest), respectively. As illustrated Figure 2, sympathetic preganglionic neurons are short and release acetylcholine onto postganglionic neurons triggering the release of mainly norepinephrine onto peripheral organs. Conversely, parasympathetic preganglionic neurons send long projections out to postganglionic neurons which are often located within the target tissue. Both pre- and postganglionic parasympathetic neurons release acetylcholine. Autonomic neurotransmitter release onto peripheral tissues is crucial for regulating key metabolic functions, which are often disrupted in chronic disease.

Sympathetic drive to brown adipose tissue (BAT) burns calories, dissipating heat, in a process called thermogenesis. Parasympathetic and sympathetic innervation of the pancreas controls glucose homeostasis through insulin release.^47^ Liver innervation regulates de novo production of glucose (gluconeogenesis), glycogen storage or breakdown, as well as lipid homeostasis.^48,49^ Sympathetic modulation of the ENS generally dampens motility, and sympathetic vasoconstrictor neurons can directly halt blood flow to the GI.^39,50^ Parasympathetic neurons stimulate pancreatic and gall bladder digestive secretions, relax sphincters, and accelerate motility.^39^

The coordinated regulation of these tissues carried out by autonomic nerves is crucial for proper delivery of nutrients into the circulation during “fighting or flight”, or to reabsorb and store nutrients during “resting or digesting.” Muller et al. beautifully demonstrated how vagal afferent neurons respond to alterations in the gut microbiota and modulate the sympathetic outflow through celiac (CG) and superior mesenteric ganglia (SMG) to control GI motility.^30^

### 2.3 Enteric neurons

**Figure 3.**
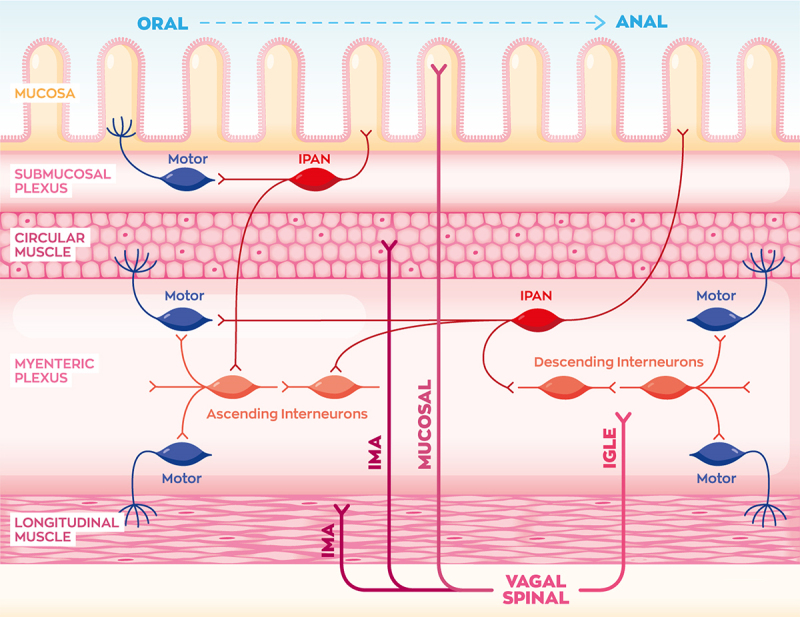
Vagal and spinal afferents are categorized based on their projections within the walls of the gastrointestinal (GI) tract. Intramuscular arrays (IMAs) terminate in the circular and longitudinal muscle, intraganglionic laminar ending (IGLEs) contact the myenteric plexus, and mucosal afferent neurons reach into the mucosa. In the enteric nervous system, intrinsic primary afferent neurons (IPANs) coordinate GI contraction and motility by sensing mechanical distension of the lumen and stimulating motor and interneurons. Motor neurons in the submucosal plexus mainly control blood flow and absorption, while myenteric interneurons and motor neurons control circular and longitudinal muscle contraction to propel food through the gut lumen. Although not shown in this image, parasympathetic and sympathetic efferent neurons also contact enteric neurons to modulate GI function.

The enteric nervous system is comprised of sensory, motor, and interneurons organized into networks or plexuses located within the gut, which are capable of operating independently of the CNS. The submucosal plexus lies between the mucosa and circular muscle, and it regulates secretion and blood flow.^39^ Enteric neurons between the circular and longitudinal muscle make up the myenteric plexus (Auerbach plexus), which controls gut motility by action on smooth muscle. Enteric sensory neurons known as IPANs (intrinsic primary afferent neurons) detect various chemicals or distension caused by a food bolus, and then coordinate the electrical activity of submucosal and myenteric neurons. Finally, interneurons link the activity of ascending and descending motor networks to allow the “little brain” of the gut to function autonomously (Figure 3).^39,51^ The enteric nervous system is also supported by local glial cells, which also respond to changes in gut microbiota signaling,^52^ but we will focus on enteric neurons in this review.

As mentioned, the enteric nervous system can work autonomously to digest a meal, but the function of these neurons is modulated by autonomic nerves, depending on internal state.^51^ The parasympathetic nervous system communicates with the enteric neurons to increase motility, secretions, and blood flow, thus aiding in “rest and digestion”. Conversely, sympathetic neurons oppose these actions to halt digestion during a “fight or flight” state of arousal.

### 2.4 Morphology of extrinsic gut innervation

As illustrated Figure 3, vagal and spinal afferent neurons can be categorized by the morphology of innervation within the layers of the gastrointestinal tract. They are classified as intraganglionic laminar endings (IGLEs), intramuscular arrays (IMAs), or mucosal innervating neurons. IGLEs project into the myenteric plexus functioning as mechanoreceptors, and IMAs innervate the circular and longitudinal muscle layers. Mucosal primary afferents project all the way into the lamina propria and intestinal villi, likely functioning as nutrient-sensing chemoreceptors.^53^ Recent work has identified mucosal afferents forming synapses with enteroendocrine cells,^54,55^ but most are thought to terminate as free nerve endings inside the villi.^40,56^ Enteroendocrine cells synapsing onto vagal afferent neurons are known as “neuropod” cells, which likely regulate feeding behavior.^55^ Future studies assessing how these “neuropod” cells sense microbial signals would be of high interest.

The different morphologies of vagal afferents appear to express distinct GPCR or neuropeptide markers. For instance, most IGLEs in the stomach express GLP-1 receptor (GLP-1 R), while small intestine IGLEs express oxytocin receptor (OxtR). Mucosal afferent neurons generally are identified by GPR65 expression,^53^ stomach mucosal neurons express calcitonin-gene regulated peptide (CGRP), and small intestine mucosal afferent neurons express VIP. Interestingly, IGLEs appear to control food intake,^57,58^ while mucosal afferents regulate glucose production in the liver.^58^ “Neuropod” cells are glutamatergic and express Peptide YY (PYY) and Cholecystokinin (CCK), so the vagal afferents synapsing with “neuropod” cells likely express the receptors for these neurotransmitters and hormones. More and more studies are beginning to elucidate the molecular mechanisms of how these different populations of neurons are relaying microbial signals to regulate host physiology,^59^ which we will now discuss.

## 3. Molecular mechanisms mediating gut microbiota-PNS communication

**Figure 4.**
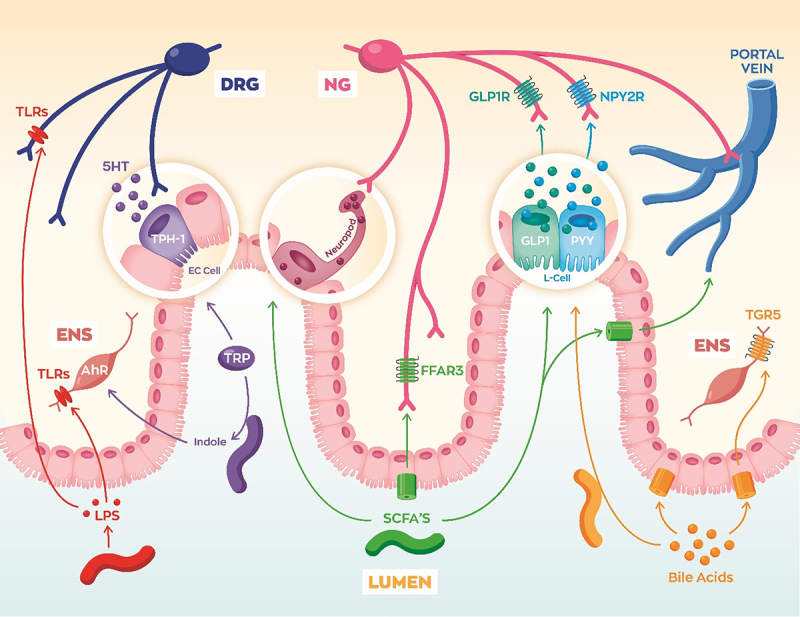
Gut microbes signal to vagal, spinal, and enteric neurons via a variety of mechanisms. Lipopolysaccharide (LPS) from gram-negative bacteria can activate neuronal toll-like receptors (TLRs). Bacteria convert tryptophan (Trp) into indole metabolites which can alter gene programming of enteric neurons via aryl hydrocarbon receptor (Ahr) signaling. Trp can also be converted into serotonin (5-HT), which is release by enterochromaffin cells (EC). Bacterial fermentation of fiber produces short-chain fatty acids (SCFAs) which can bind free fatty acid receptor 3 (FFAR3). SCFAs can also trigger L-cells to release neuropeptides glucagon-like peptide 1 (GLP-1) and peptide YY (PYY). Gut microbe production of secondary bile acids binds Takeda G-protein receptor 5 (TGR5) on L-cells to trigger GLP-1 and PYY release. Secondary bile acids can also signal to TGR5 on enteric neurons to regulate motility.

As previously mentioned, the signaling between the gut microbiota and peripheral neurons occurs through three interacting pathways. Specialized endothelial cells in the lining of the gut are called enteroendocrine cells (EECs) and they represent a major part of the hormonal pathway. EECs respond to chemical byproducts and signaling molecules released by the gut bacteria, and in turn they release neuropeptides capable of activating vagal sensory neurons or circulating to directly target effector tissues (Figure 4). The receptors for EEC hormones are expressed in vagal sensory neurons innervating the gut, and various physiological processes are controlled via this pathway such as gastric emptying, gut motility, insulin release, satiety, and hunger.^31,51,57,58,60,61^ We will breakdown different molecules secreted, modified, or stimulated by gut microbes and discuss the mechanisms by which they interact with peripheral neurons. We will also briefly discuss the reverse interaction where peripheral neurons release neurotransmitters and neuropeptides to modulate microbial activity directly or via immune activation.

### 3.1 Lipopolysaccharide (LPS) and Lipoteichoic Acid (LTA)

Lipopolysaccharide and lipoteichoic acid are surface proteins found on gram-negative and gram-positive bacteria, respectively. LPS and LTA are considered pathogen-associated molecular patterns (PAMPs) because they can trigger innate immune responses through toll-like receptors (TLRs). Circulating LPS levels are altered based on diet,^62^ with increased levels in high fat diet (HFD) rodent models,^63,64^ and in people with a subset of IBS (diarrhea-predominant).^65^ LPS levels also correlate with weight gain,^62^ and chronic LPS injections can induce adipose macrophage infiltration^66^ and weight gain.^63^

Along with immune cells, enteric, spinal, and vagal neurons express toll-like receptors (TLRs). Disrupted TLR signaling in peripheral neurons may contribute to deficits in enteric nervous development, gut motility, immunity, and visceral perception (Figure 4). LPS binds to toll-like receptor 4 (TLR4) and LTA binds TLR2. TLR2 expressed in spinal sensory neurons and activation can alter the reflex release of neuropeptides capable of increasing cytokines.^67^ TLR4 is expressed in vagal neurons which play a key role in the inflammatory reflex where the brain senses potential proinflammatory signals and regulates the immune responses.^67,68^ This is accomplished by vagal efferent release of acetylcholine onto immune cells in target tissues which can dampen the local immune response. Additionally, vagal TLR4 signaling mediates LPS-induced release of the neuropeptide calcitonin gene-regulated peptide (CGRP).^69^ CGRP is a vasodilatory neuropeptide released by spinal and vagal afferent neurons, as well as enteric neurons, which contributes to inflammation resolution and pain.^70^

Enteric neurons express both TLR2 and TLR4. Surprisingly, TLR2 agonists have been shown to promote neurogenesis and restore myenteric neurons in the colon of GF mice and in mice after antibiotic-induced depletion of microbiota.^36^ TLR4 signaling also promotes enteric neuron survival and regulates proper gastrointestinal motility.^71^ While TLR signaling appears to be crucial for proper GI development and function, unresolved TLR signaling likely contributes to pain from infection or dysbiosis.^72^ Future studies likely need to segregate developmental vs. acute signaling roles for TLRs. Furthermore, many studies do not distinguish direct effects of TLR signaling in neurons from immune cell TLR signaling, which indirectly activates peripheral neurons.

### 3.2 Tryptophan metabolites

Tryptophan (Trp) is an essential amino acid implicated in various diseases such as IBS, metabolic disease, and potentially neurological disease as well.^73–75^ Trp is metabolized into three major pathways: serotonin (5-HT), kynurenine, and indole. Humans and rodents express enzymes for the conversion of tryptophan into serotonin and kynurenine metabolites, but these pathways are highly influenced by gut microbes, as reviewed extensively by Agus et al.^75^ Kynurenic acid activates GPR35,^76^ which is highly expressed in vagal sensory neurons,^77^ although the function of vagal GPR35 has yet to be directly demonstrated. Trp conversion into indole metabolites mostly requires bacterial enzymes. Indoles can freely diffuse through enterocytes and bind the aryl hydrocarbon receptor (Ahr), which is a nuclear receptor controlling gene expression. Immune Ahr signaling regulates intestinal inflammation, however different studies contradict in whether Ahr is pro- or anti-inflammatory.^78^

Focusing on PNS Ahr signaling, Obata and colleagues beautifully demonstrated that gene expression of enteric neurons is regulated by local gut bacteria, and the transcriptional programming varies along the intestine due to the bacteria colonized in that region.^79^ Germ-free (GF) and mice with deletion of enteric neuron Ahr show similarly reduced intestinal transit time compared to specific-pathogen-free (SPF) and Ahr expressing controls. Viral vector expression of Ahr was able to partially rescue disrupted intestinal transit in antibiotic treated mice. Interestingly, the authors suspected that depletion of microbiota-induced serotonin^32^ likely explained the limited rescue of intestinal transit after viral overexpression of Ahr.

### 3.3 Serotonin (5-HT)

Gut bacteria regulate the production and release of serotonin (5-HT) and 5-HT signaling is disrupted in GF mice,^80,81^ obesity models,^82^ as well as IBS patients.^83^ Enterochromaffin cells (EC) are the most common enteroendocrine cell in the gut, distributed throughout the entire GI tract, and they produce approximately 90% of the 5-HT in our body. While the gut microbiota does not produce 5-HT directly, SCFAs and secondary bile acids from the gut microbiota can stimulate 5-HT production and release.^80^ Proper 5-HT production and signaling is crucial for enteric neuron development, maturation, and adult function.^33,84^ De Vadder et al. found that colonizing GF mice with conventional gut bacteria restored serotonin levels and induced the proliferation of enteric neurons. They demonstrated that the 5-HT_4_ receptor was key to enteric neuron survival by showing that a specific agonist for this receptor was able to restore ENS innervation and intestinal transit time.^33^ 5-HTR_3_ is expressed in intrinsic primary afferent neurons (IPANs), which are crucial for converting chemical and mechanical stimuli from the gut lumen to the submucosal and myenteric plexus for proper GI motility, secretion, and blood flow.^85^ Serotonin can also bind receptors expressed in vagal neurons to slow gastric emptying and delay motility.^84^ Enterochromaffin cells also form synaptic connections directly with spinal neurons, similar to “neuropod” cells. This connection may serve as a mechanism to fine tune motility, intestinal inflammation, and possibly visceral pain.^54^

### 3.4 Gamma-aminobutyric acid (GABA)

Several *Lactobacillus* and *Bifidobacterium* species are capable of producing the inhibitory neurotransmitter gamma-aminobutyric acid (GABA).^86^ They do so by converting glutamate into GABA via the enzyme glutamate decarboxylase (GAD), which functions to reduce the local acidity.^87^ While GAD-expressing bacteria apparently benefit from the lower pH, the host has clearly evolved to respond to the GABA by-product via a variety of mechanisms. GABA_B_ receptors are GPCRs expressed in enteric, spinal, and vagal afferents,^88,89^ and activation of this receptor is generally inhibitory.^90^ For instance, one group found that a GABA-producing probiotic reduced spinal neuron excitability in a rat model of visceral hypersensitivity. Gavage of a Bifidobacterium species lacking GABA-producing enzymes failed to reduce neuronal excitability in their model.^24^ Activation of GABA_B_ receptors on vagal neurons reduces the sensitivity to mechanical stimuli such as stretch or brushing.^89,91^ Additionally, vagal GABA_B_ signaling controls gastric emptying, motility, and digestive secretions making it an interesting therapeutic target for functional GI disorders.^90^ Fecal microbiota transplantation (FMT) from lean donors dramatically increased circulating GABA levels in individuals with metabolic syndrome, which may have contributed to improved insulin sensitivity.^92^

### 3.5 Bile acids

Primary bile acids, cholic acid (CA) and chenodeoxycholic acid (CDCA), are produced by the liver and secreted into the small intestine to emulsify dietary fat and cholesterol, which is critical for digestion and nutrient absorption. Bacteria residing in the gut can modify bile acids into lithocholic acid (LCA) and deoxycholic acid (DCA) which are known as secondary bile acids. While these bile acids play an essential role in dietary lipid absorption, and they also function as signaling molecules for farnesoid X receptor (FXR) and Takeda G-protein receptor 5 (TGR5). TGR5 is a GPCR expressed in spinal neurons,^93^ enteric neurons,^94^ and vagal neurons,^95^ and is primarily activated by LCA.

In vagal afferent neurons, TGR5 is co-expressed with cholecystokinin (CCK) receptor+ neurons which are known to regulate feeding behavior. One study in rats demonstrated that bile acids reduce food intake, dependent upon TGR5 expression in vagal neurons. They also showed that TGR5 and CCK synergistically activate nodose neurons to reduce food intake.^95^ TGR5 activation induces calcium (Ca^2+^) responses in L-cells and stimulates their release of GLP-1,^96^ which regulates glucose homeostasis and feeding behavior via vagal and/or hormonal routes.^97^ TGR5 is also expressed in the hypothalamus, and bile acids can circulate directly to the brain and prevent diet-induced obesity.^98^ LCA and DCA also activate TGR5 in enteric neurons and enterochromaffin cells (EC) in the colon to control motility.^99,100^

### 3.6 Short chain fatty acids (SCFAs)

SCFAs are monocarboxylic acids (acetate, propionate, butyrate, and valerate) produced by fermentation of fiber by various genera of bacteria including *Lactobacillus, Bifidobacterium, Prevotella*, and *Bacteroides*.^101^ Interestingly, these bacterial metabolites exert pleiotropic effects on the host via multiple signaling mechanisms and are an intriguing signal between the gut and the brain.^102–104^ Focusing on their signaling roles in peripheral neurons, SCFA-binding G-protein coupled receptors are expressed in various peripheral ganglia. For instance, free fatty acid receptor 3 (FFAR3) is expressed in several sympathetic ganglia, enteric neurons, vagal ganglia, celiac/superior mesenteric, and dorsal root ganglia.^77,105–107^ Distinct roles have been suggested for FFAR3 in spinal, autonomic, and enteric neurons in regulating intestinal gluconeogenesis,^32,104^ heart rate,^106^ energy expenditure,^108^ and food intake.^59^ FFAR3 signaling also influences fetal nervous system development. The gut microbiota is established after birth, however production of SCFA from the pregnant mother can signal to the developing fetus via FFAR3 and promote sympathetic neuron innervation and neurite outgrowth.^38^ Ultimately, lack of FFAR3 signaling in utero predisposed the mice to HFD-induced obesity and metabolic syndrome via reduced energy expenditure.^38^ Using a novel FFAR3 flox mouse model, we found that vagal FFAR3 signaling regulates short-term feeding behavior in adults.^59^ More studies utilizing cell-type specific and inducible knockout models are necessary to clearly elucidate adult vs. developmental signaling roles for peripheral neurons in sensing fiber fermentation by the gut microbiota.

SCFAs also bind Olfactory receptor 78 (Olfr78) expressed in a subset of vagal sensory neurons,^57^ however, supraphysiological levels are required for activation. Physiologically, vagal Olfr78 likely is predominantly activated by lactate, which regulates respiration via innervation of the heart and lungs.^109^ FFAR2 and hydroxycarboxylic acid receptor 2 (HCAR2) also bind SCFAs, but to our knowledge, no studies have clearly defined neuronal populations expressing these receptors.

### 3.7 Cocaine-amphetamine regulated transcript (CART)

Cocaine-amphetamine regulated transcript (CART) is a neuropeptide expressed in vagal and enteric neurons, as well as in the CNS. Vagal neuron release and expression of CART is modulated based on feeding status via leptin and CCK.^110,111^ CART+ enteric neurons are modulated by the microbiota and regulate glucose homeostasis via direct communication with the pancreas and liver.^112^ Muller et al. also identified other ENS transcripts dysregulated in GF mice, including somatostatin (SST) and agouti-related peptide (Agrp),^112^ which warrants further investigation.

### 3.8 Substance P and calcitonin gene-regulated peptide (CGRP)

Excitation of spinal and vagal afferent neurons can induce the release of neuropeptides such as Substance P and CGRP, which control blood flow and immune cell function,^113^ while possibly possessing antimicrobial properties.^114^ Both peptides are also implicated in visceral and peripheral pain.^115^ Increased levels of Substance P have been seen in the colon after antibiotic treatment^21^ and in colitis models.^116^ As previously mentioned, vagal neurons release CGRP in response to TLR4 activation,^69^ but more work is needed to determine the physiological consequences of this signaling.

## 4. Gut microbiota to PNS communication as a therapeutic target

**Figure 5.**
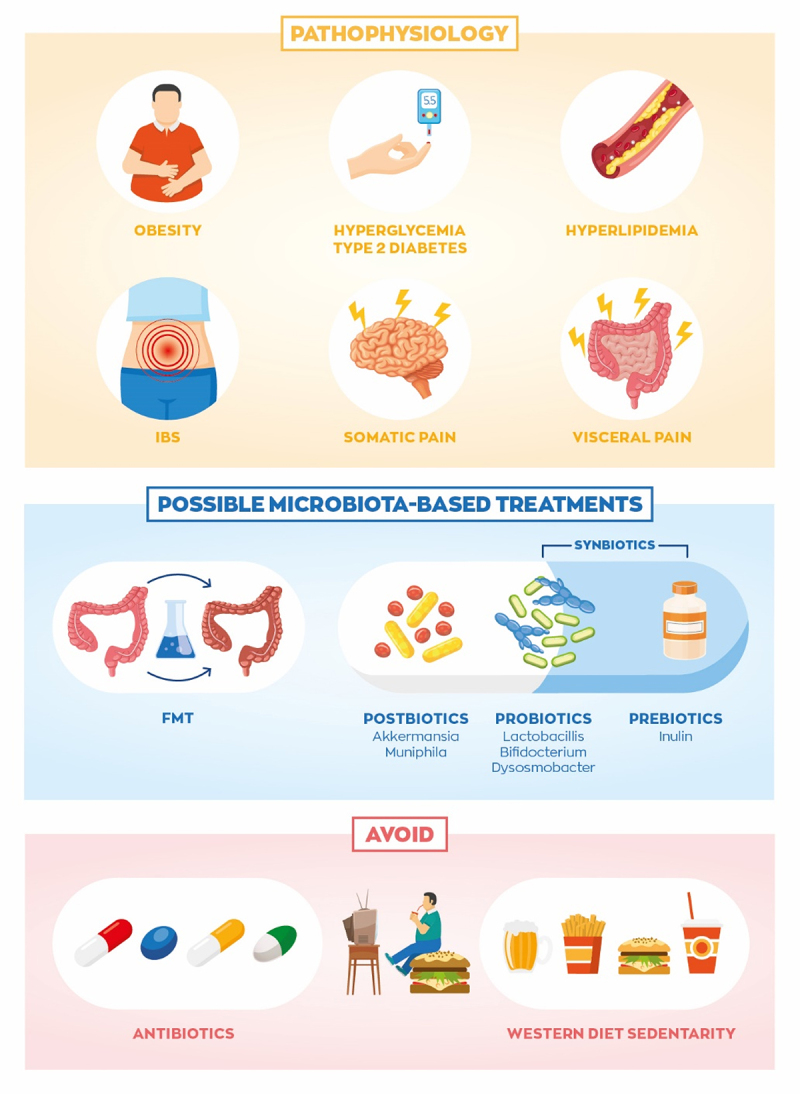
Disrupted gut microbe to peripheral nervous system signaling can lead to obesity, irritable bowel syndrome, and associated comorbidities. Microbiota-targeted therapies such as fecal microbiome transplantation (FMT), postbiotics, probiotics, prebiotics, and combinations may help improve obesity- and IBS-associated complications. Additionally, avoiding antibiotics and western diet may prevent progression of these diseases.

Numerous lines of evidence point to various members of the gut microbiota as contributors in chronic diseases, and microbiota-targeted therapies may provide benefits. Sensory and autonomic nerves innervating the GI tract are sensitive to infection, inflammation and local metabolites which can lead to changes in energy and glucose homeostasis, motility deficits and hypersensitivity commonly seen in IBS, functional dyspepsia, diabetes, or obesity.^117,118^ Microbiota-targeted treatments for these ailments come in the form of prebiotics, probiotics, postbiotics, fecal microbiota transplantations (FMT), and antibiotics (Figure 5). Prebiotics consist of nutrients directly targeted to feed or promote certain bacterial growth. For instance, dietary fibers like inulin are fermented by our gut bacteria and can provide therapeutic benefits to people who are overweight,^119^ diabetic,^120^ or suffering from IBS.^121^ Probiotics and postbiotics are viable or dead cultures of bacteria, respectively. Combinations of pre- and probiotics are referred to as synbiotics. FMT is a less specific approach where healthy donor bacteria are transferred to a diseased recipient in the hopes of restoring the perturbed microbiota back to healthy form. Different studies and treatment strategies utilize various combinations of pre-, pro-, and/or or postbiotics with antibiotics and/or FMT.

### 4.1 Obesity and accompanying comorbidities

Obesity is defined by a body mass index (BMI) of 30 kg/m^2^ or higher, and is associated with increased risk of several comorbidities, particularly heart disease and diabetes. Although studies have been inconsistent in identifying a clear microbiome signature associated with obesity pathogenesis, many recent studies have found effective microbiota-targeted therapies, and we are beginning to uncover some of the signaling mechanisms. For instance, probiotic supplementation of the commensal *Dysosmobacter welbionis* induced weight loss in mice, likely via activation brown adipose thermogenesis via unknown mechanisms^25^. The authors speculated that production of the SCFA butyrate was the driver of increased energy expenditure. SCFAs target autonomic circuits regulating glucose homeostasis, motility, feeding behavior, and energy expenditure, which offers promise for anti-obesity therapies. Li et al. demonstrated the thermogenic capability of butyrate to restore energy expenditure after antibiotics depletion.^122^ SCFAs directly activate FFAR3 in sympathetic neuron to increase energy expenditure^106^ and vagal sensory neuron to reduce food intake,^31,59^ thus bidirectionally improving energy balance. Therefore, therapies increasing gut microbe SCFA production could help obese individuals lose weight.^123^ In addition to SCFAs directly activating autonomic neurons, they can also regulate the release of EEC hormones such as GLP-1^124^ and GIP.^125^ These hormones induce potent effects on the host to decrease food intake^126^ and stimulate insulin release,^127^ resulting in a huge effort to produce GLP1 and GIP mimetics for obesity and diabetes.^97,128^ Endogenous release of these peptides could also be therapeutically targeted via modulation of the gut microbiota. Conversely, other studies show that SCFAs increase dietary energy harvest and weight gain, leading to disrupted glucose homeostasis.^129,130^ Finding the right dose and method of delivery may be necessary to establish SCFAs as a safe and effective dietary supplement.

One major complication of obesity is dysfunctional glucose homeostasis and increased comorbidity of type II diabetes. FMT, probiotic supplements, and dietary regiments have all been shown to improve host glucose homeostasis.^92,131,132^ A series of brilliant studies from Dr. Cani’s lab demonstrated *Akkermansia municiphila* to be beneficial for alleviating obesity-associated glucose and lipid disruptions in rodents^20^ and humans.^18^ The same group later discovered that a membrane protein that activating TLR2 was responsible for the metabolic improvements, and viable bacteria was not needed.^19^ Thus, *Akkermansia muciniphila* is a promising postbiotic supplement to improve metabolic disturbances in obesity. Further work examining the signaling between *Akkermansia* and TLR2 in peripheral neurons may provide interesting insights into future therapies for pain and motility issues, as well.

Pain also presents as a common feature in obesity and type II diabetes,^133^ which primarily manifests as somatic pain in the periphery, rather than in the viscera. In a case report, a diabetic woman received FMT from a healthy donor which improved glucose levels and improved indices of pain.^134^ FMT from lean mice to obese mice also shows efficacy in improving peripheral hypersensitivity caused by a western diet, independent of glucose lowering or weight loss.^135^ The downfall of FMT-based therapies is the lack of insight provided into how the disease symptoms are alleviated, or which bacteria are providing benefits. More work is needed to elucidate the molecular mechanisms linking obesity-associated pain and the microbiota. While we are still discovering the precise signaling processes involved, microbiota-targeted therapies show great promise for obese individuals by improving glucose intolerance, insulin sensitivity, satiety, appetite, and weight management.

### 4.2 Irritable bowel syndrome

Irritable bowel syndrome (IBS) is currently the most common functional GI disorder affecting between four and ten percent of the population worldwide, based on conservative estimates.^136^ Rates of IBS are potentially even higher in the western world, and a “western diet” high in fat and sugar may increase IBS risk^137^ (Figure 5). The underlying causes of IBS remain a mystery, however symptoms often develop after an acute bout of bacterial gastroenteritis. Later, hallmark features develop, including chronic low-grade inflammation, gastrointestinal motility issues and visceral pain.^136^ Thus, the interaction between peripheral neurons and the gut microbiota is highly implicated and serves as a promising therapeutic target.^11,138,139^ IBS can be divided into subtypes depending on the symptoms including bloating, diarrhea or constipation, cramping, and sometimes mood disruptions.^6^ Again, many of these symptoms correlate with disrupted PNS signaling, whether it be aberrant afferent signaling contributing to intestinal discomfort,^41,139^ disrupted enteric neuron excitability,^140^ or dysregulated autonomic signaling to the gut.^26,141,142^

Interestingly, FMT improved patient outcomes in several IBS trials, while actually worsening symptoms in other studies.^143^ Given the heterogeneity of symptoms across different types of IBS, a one-size fits all therapy is not likely realistic. Finding ways to individualize therapies for each patient’s unique symptoms and microbiota may be necessary to provide effective treatment.^143^ As opposed to obesity-associated peripheral pain, IBS often presents with intestinal pain. In animal models, antibiotic treatment causes visceral hypersensitivity,^21,144^ which can be prevented by probiotic supplementation of *Lactobacillus*.^21,22^ Several *Lactobacillus* and *Bifidobacterium*^23,25^ probiotics have been developed and show promise for the treatment of visceral pain in humans, as well. While the mechanisms have not been clearly demonstrated, *Lactobacillus* and *Bifidobacterium* produce GABA and SCFAs,^24,86,88,102,135^ and these molecules appear to exert inhibitory effects on enteric and spinal neurons,^24,86,88^ which may underly the alleviation of painful symptoms. Some studies show the prebiotic inulin improving constipation in people with IBS,^145^ however inulin can also cause gas. Combining another prebiotic, psyllium, counteracted this side effect of inulin.^146^ Numerous lines of evidence also point to disrupted tryptophan metabolism in IBS.^75,80^ IBS patients also exhibit psychiatric comorbidities, such as heightened stress response mediated by the hypothalamic-pituitary-adrenal (HPA) axis.^26,142^ There is a vicious cycle of bi-directional dysfunction of the gut brain axis where disruptions in microbiome signaling cause aberrant sensory signals, but perturbations in autonomic outflow can exacerbate intestinal inflammation and dysbiosis. Breaking this cycle through personalized treatments targeting gut microbe to peripheral neuron interactions may provide relief to the many people suffering from IBS.

## 5. Obstacles, conclusions and future directions

There is still large gap in the knowledge of molecular mechanisms driving the interaction between the gut microbiota and host physiology. The shortfall of microbiome sequencing approaches is that there likely exist a high level of redundancy in the genes and metabolic pathways across different genus of bacteria. Conversely, different species within the same genus may express distinct genes that affect the microbiota landscape or directly signal to the host in a unique way. Furthermore, it is difficult to identify a clear microbial signature driving disease or physiological process when the microbiome differs in individuals across different regions. The functional output of an individual’s microbiota may provide more physiological information than purely microbiome composition. Thus, future studies utilizing proteomic approaches to determine alterations in metabolites or peptides produced by the gut microbiota may prove very insightful.

Although many promising microbiota therapies have been demonstrated in rodent studies, translating to human application has been more difficult for several reasons. Studies tend to use only male mice, yet sex is a key genetic variable in metabolism and GI function,^147–149^ and IBS prevalence may be higher in females.^136^ Additionally, laboratory rodents live in excessively sterile environments that prevent the animals from proper immune^150,151^ and nervous system development,^152^ rendering them less relevant as model organisms. Paradoxically, dirtier laboratory environments may yield results that offer more translational value to humans.^151^ Another obstacle preventing us from understanding PNS/microbiota signaling has been identifying the specific cell populations involved. The gut is comprised of complex interactions of neurons, glia, immune, and other cells making it difficult determine the culprit of disease pathogenesis. Continuing with cell-type specific studies in relevant disease models will help us develop more effective treatments.

Despite these obstacles, it is becoming increasingly clear that interaction between gut microbiota and our nervous system is critical for our health and wellness. The microbes in our gut produce a variety of molecules capable of signaling to our peripheral neurons. From the very beginning of life in the womb, the mother’s gut microbes are already regulating fetal nervous system development. Disruptions in gut microbiome composition after infection, antibiotic use, dietary changes, and other environmental influences contribute to dysregulated host functions mediated by peripheral neurons. Continuing our understanding of the molecular mechanisms by which the host senses and responds to the gut microbiota signaling will guide improved development of therapies to combat the epidemics of obesity and gastrointestinal disease.
